# Optics and Biologic Connectedness

**DOI:** 10.3201/eid1103.AC1103

**Published:** 2005-03

**Authors:** Polyxeni Potter, comments to authors

**Affiliations:** * Centers for Disease Control and Prevention, Atlanta, Georgia, USA

**Keywords:** Art and science, emerging infectious diseases, Seurat, pointillism, impressionism, optics, connectedness, diphtheria, Ebola

**Figure:**
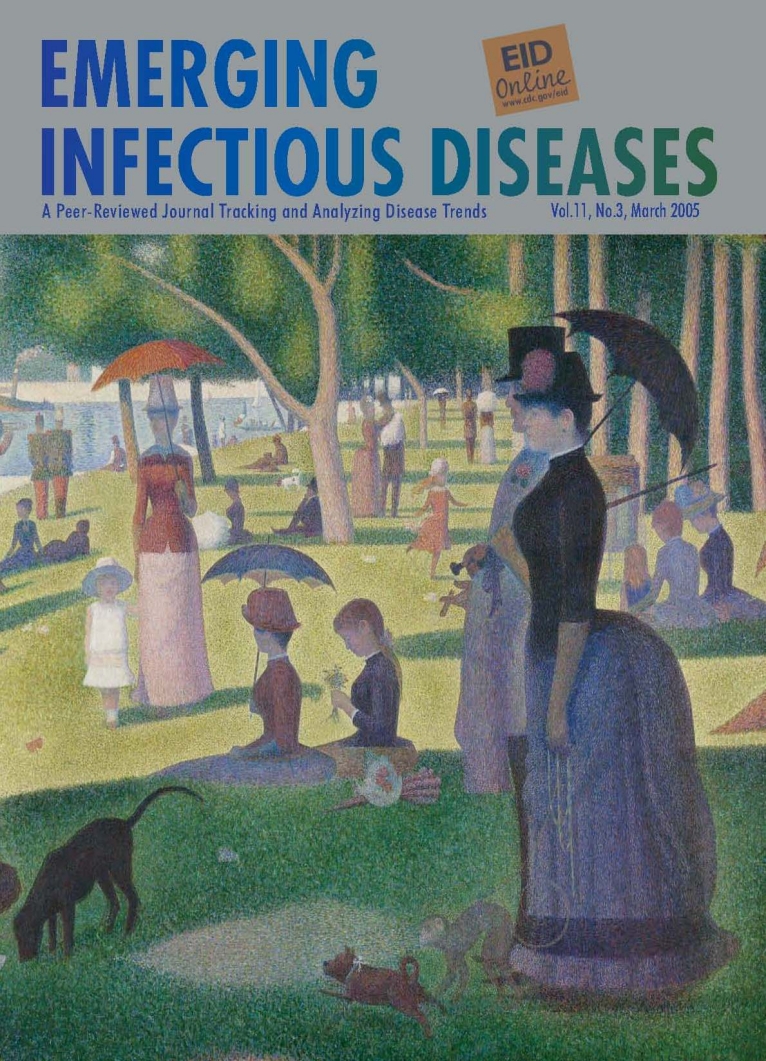
**George Seurat (1859–1891). Sunday Afternoon on the Island of La Grande Jatte, detail (1884–86).** Oil on canvas (2.08 m x 3.08 m). The Art Institute of Chicago

"…It is this passion for beautiful colors that makes us paint as we do… and not the love of the 'dot,' as foolish people say," wrote painter Paul Signac in his journal (*1*). He was defending the art movement started by his good friend and fellow artist George Seurat and built upon by Signac himself, Camille Pissarro, and others. This movement, divisionism or pointillism, was Seurat's artistic contribution during a brief but extraordinary life.

Parisian from a middle-class family, tall, and handsome, Seurat enjoyed a comfortable life and proper education. He showed early talent for drawing, studied sculpture, and attended the prestigious École Des Beaux-Arts. A competent photographer, he became interested in the workings of light, particularly in black-and-white images. This interest grew as he studied optics and the processes at work on the silver particles of photographic film (*2*). During his art studies, particularly under the tutelage of a student of Ingres, he came to believe in a systematic approach to art.

Nicknamed "le notaire" (the notary) for his immaculate attention to his appearance, Seurat was temperamentally suited for a scientific approach to art (*3*). Idiosyncratically bent toward order and control and gifted with formidable observational skills, patience, concentration, and painstaking adherence to detail, he embarked on a style of painting based on color and structure that was cerebral and calculated.

Like the impressionists, Seurat was interested in the relationship between natural light and the application of paint, only he wanted to create an impression not on the canvas but in the mind of the viewer. Influenced by the work of French chemist Michel-Eugène Chevreul (1786–1889), he believed that next to each other, colors appear as dissimilar as possible, both in optical composition and tonal value (*4*). Seurat's color theory, in which the viewer plays a key role in perception, influenced the development of modern art.

His artistic goal, Seurat once said, was to show "modern people, in their essential traits, move about as if on friezes, and place them on canvases organized by harmonies of color, by directions of the tones in harmony with the lines, and by the directions of the lines" (*5*). In his best known work, images are tightly structured as if on a grid, the figures systematically placed in relation to each other in permanent, non-negotiable arrangements. Pure color is used directly from the tube, in static "points" clearly separate but intended to merge in the viewer's eye, producing a confluent image brighter than any achieved with brushstrokes. Like many scientific experiments, Seurat's daring process had unexpected results. The points remained visible, akin to tesserae in a mosaic, but produced a shimmering translucent effect (*5*).

A Sunday Afternoon on the Island of La Grande Jatte, on this month's cover of Emerging Infectious Diseases, is Seurat's masterpiece and one of the best-known works of the 19th century. The placid scene in an island park on the Seine shows a local crowd during a moment of leisure outdoors. Seurat's version of this commonplace event is revolutionary. As figures register in the viewer's eye, they seem suspended in mid-moment, levitating yet permanently fixed. Prototypes rather than likenesses, they represent workers in shirt sleeves, fashionable couples, children at play, soldiers in uniform. Seurat did not dwell on their faces, nor did he offer anything but their frontal or profile forms—classical, refined, distinct, balanced, and frozen in time. The iconic setup, like backdrop in a period drama, impassionedly places people, animals, and objects in a suddenly interrupted scene, creating a spellbinding visual effect.

As much interested in the science as in the art of painting, Seurat used figures as scene building blocks. Elegantly curved and grouped in harmonious ensembles, the figures are isolated from each other and detached from the beauty around them. And like separate dots of color, they do not fully blend, their shimmering presence only a means to a perfect artistic end.

Seurat's own life embodied the personal isolation seen in Sunday on La Grande Jatte. Even though surrounded by friends and supported by family, he was intensely private, even secretive, about his affairs. His parents did not know that he had a child until he was taken ill, possibly with diphtheria (*6*). He died precipitously at age 31, while hard at his innovative work. Signac encapsulated his friend's achievement, "He surveyed the scene and has made these very important contributions: his black and white, his harmony of lines, his composition, his contrast and harmony of color, even his frames. What more can you ask of a painter?" (*1*).

Seurat was not interested in the emotional or evolutionary connectedness of the crowd in La Grande Jatte. The nannies, belles and beaux, the playful pet monkey, even the stray dog foraging picnic crumbs in the foreground, are locked into themselves. Had Seurat been interested in biologic rather than optical accuracy, he might have ventured beyond visual perception of the crowd on the lawn. And between the dots, he might have found invisible connectedness, the glue that binds humans, monkeys, stray dogs, vegetation. Impervious to optics and inaccessible to the naked eye, biologic connectedness abounds.

Around the world, as in La Grande Jatte, scrounging animals share the landscape with humans. Along with scraps of food, they gather data that properly transcribed can be valuable. In African forest villages, loose dogs living near hunters and eating dead animals become exposed to Ebola and carry antibodies to the virus. Their destinies intertwined with ours in a way inaccessible to Seurat, the dogs may become predictors of human disease as their serologic status signals the presence of virus in the community (*7*).
